# A Meta-Analysis of Using Protamine for Reducing the Risk of Hemorrhage During Carotid Recanalization: Direct Comparisons of Post-operative Complications

**DOI:** 10.3389/fphar.2022.796329

**Published:** 2022-02-25

**Authors:** Yongli Pan, Zhiqiang Zhao, Tao Yang, Qingzheng Jiao, Wei Wei, Jianyong Ji, Wenqiang Xin

**Affiliations:** ^1^ Department of Neurology, Weifang Medical University, Weifang, China; ^2^ Department of Neurosurgery, Heji Hospital Affiliated Changzhi Medical College, Changzhi, China; ^3^ Second Department of Internal Medicine, Gucheng Country Hospital, Shijiazhuang, China; ^4^ Department of Neurology, Mianyang Central Hospital, Mianyang, China; ^5^ Department of Neurosurgery, Liaocheng People’s Hospital, Liaocheng, China; ^6^ Department of Neurosurgery, Tianjin Medical University General Hospital, Tianjin, China

**Keywords:** protamine, carotid recanalization, meta-analysis, carotid stenosis, hemorrhage - cerebral

## Abstract

**Background:** Protamine can decrease the risk of hemorrhage during carotid recanalization. However, it may cause severe side effects. There is no consensus on the safety and efficacy of protamine during surgery. Thus, we conduct a comprehensive review and meta-analysis to compare the differences between the protamine and the no-protamine group.

**Method:** We systematically obtained literature from Medline, Google Scholar, Cochrane Library, and PubMed electronic databases. All four databases were scanned from 1937 when protamine was first adopted as a heparin antagonist until February 2021. The reference lists of identified studies were manually checked to determine other eligible studies that qualify. The articles were included in this meta-analysis as long as they met the criteria of PICOS; conference or commentary articles, letters, case report or series, and animal observation were excluded from this study. The Newcastle-Ottawa Quality Assessment Scale and Cochrane Collaboration’s tool are used to assess the risk of bias of each included observational study and RCT, respectively. Stata version 12.0 statistical software (StataCorp LP, College Station, Texas) was adopted as statistical software. When *I* ^2^ < 50%, we consider that the data have no obvious heterogeneity, and we conduct a meta-analysis using the fixed-effect model. Otherwise, the random-effect model was performed.

**Result:** A total of 11 studies, consisting of 94,618 participants, are included in this study. Our analysis found that the rate of wound hematoma had a significant difference among protamine and no-protamine patients (OR = 0.268, 95% CI = 0.093 to 0.774, *p* = 0.015). Furthermore, the incidence of hematoma requiring re-operation (0.7%) was significantly lower than that of patients without protamine (1.8%). However, there was no significant difference in the incidence of stroke, wound hematoma with hypertension, transient ischemic attacks (TIA), myocardial infarction (MI), and death.

**Conclusion:** Among included participants undergoing recanalization, the use of protamine is effective in reducing hematoma without increasing the risk of having other complications. Besides, more evidence-based performance is needed to supplement this opinion due to inherent limitations.

## Introduction

Ischemic stroke accounts for the mortality of approximately more than ten million lives per year all over the world (Yip et al., 2016), and it is an important public health concern. Considerable research evidence demonstrates that the prevalence of carotid stenosis is about 7% (Dharmakidari et al., 2017), which is becoming an important public health issue (Abbott et al., 2015). Carotid endarterectomy (CEA), performed to prevent embolus, is considered a conventional treatment (Howell, 2007). Carotid artery stenting (CAS), a minimally invasive procedure (Spiliopoulos et al., 2019), has emerged as an effective treatment modality for carotid artery stenosis (Setacci et al., 2018). Although these two surgical interventions have improved the prognosis of ischemic stroke, they may carry hemorrhage as a severe complication (Spence et al., 2016). Heparin is a robust anticoagulant used routinely during both CEA and CAS surgeries to avoid thromboembolic complications (Lynch and Kavanagh, 2016; Sokolowska et al., 2016). After such surgeries, some surgeons advocate the adoption of protamine to achieve a systemic anticoagulant effect to decrease the risk of hemorrhage (Liang et al., 2021). Protamine is known to be arginine-rich, making it a positively charged protein (Bakchoul et al., 2016), and is an approved drug by the Food and Drug Administration (FDA). Despite its neutralization action, protamine may cause severe side effects such as systemic hypotension, anaphylactic reaction, pulmonary hypertension, and tissue damage of the lungs, kidneys, and red blood cells (Sokolowska et al., 2016). Hence, the use of protamine can have a significant difference in short-term and long-term morbidity and mortality (Al-Kassou et al., 2020). As one study reviewed 10,059 CEAs performed in 9,260 patients from 2003 to 2012, protamine use remained stable from 2003 through 2007 at 43%. Then, there was a significant increase in protamine use to 52% from the beginning in January 2008 (Patel et al., 2013). Theoretically, protamine can bind with the glucosaminoglycan of heparin to form a stable complex, which, in turn, suppresses the activity of antithrombin, herein counteracting the anticoagulant effect of heparin and achieving the effect of hemostasis. Some surgeons advocate the routine use of protamine to minimize bleeding complications, whereas some others avoid heparin reversal to minimize the risk of stroke through thrombus formation on the endarterectomy surface of the artery (Cho et al., 2012). Therefore, the purpose of this study is to evaluate the safety and efficacy of protamine to reduce the risk of hemorrhage during carotid recanalization.

## Materials and Methods

### Literature Search Strategy

We systematically obtained literature from Medline, Google Scholar, Cochrane Library, and PubMed electronic databases. We utilized controlled vocabulary to build the search terms such as the National Library of Medicine in this study. All four databases were scanned from 1937 when protamine was first adopted as a heparin antagonist until February 2021 for the keywords of protamine, carotid endarterectomy, and carotid artery stenosis in combination with Boolean logic (Jaques, 1973). The specific search strategy is shown in Table 1. After the original search, the relevant studies and their references were searched manually by two authors. Beyond that, all references to previous reviews and related clinical trials were manually checked to identify potential publications that were not included in our electronic search results.

**TABLE 1:** The specific search strategy.


Carotid stenosis OR carotid artery stenosis OR carotid disease OR carotid artery disease
AND
CAS OR carotid artery stenting OR carotid angioplasty OR carotid stenting OR CEA OR carotid endarterectomy OR endarterectomy OR carotid surgery OR carotid revascularization
AND
Protamine

### Inclusion and Exclusion Criteria

Studies are considered eligible if they fulfilled the predefined inclusion criteria: (1) population: participant with carotid stenosis; (2) intervention: all patients strictly undergoing carotid recanalization; (3) comparison intervention: use of protamine to no-protamine group; (4) outcome measures: one or more of the following outcomes were reported: complications of wound hematoma, hematoma requiring re-operation, wound hematoma with hypertension, transient ischemic attacks (TIA), myocardial infarction (MI), stroke, and death; and (5) official published prospective and retrospective studies in English.

The exclusion criteria are listed as follows: (1) conference or commentary articles and letters, (2) atypical patients and outcome data, (3) case report and case series, and (4) animal observation.

### Data Extraction and Outcome Measures

Data were extracted by using a form prepared in advance and from the eligible researchers. Each relevant study was independently captured by two authors for the following essential details: the first author of the study, publication year, type of study, quality assessment, endpoints, and study characteristics including the number of populations in total, mean age, and gender ratio, among others. All disagreements were discussed until a final decision is reached. The primary study endpoint measurements are relevant to hemorrhagic damage including wound hematoma, hematoma requiring re-operation, and hematoma with hypertension, and secondary endpoints were the composite of ischemic injuries including stroke, TIA, and MI. Herein, in primary endpoints, all hematoma was defined as wound hematoma. In secondary endpoints, stroke was defined as 1 or more of the following: (1) an increase in the National Institute of Health stroke scale (NIHSS) score of >4 points from pre-stroke score; (2) an increase in the MRS score of >2 points from the pre-stroke score; or (3) stroke leading to a modified Rankin scale (MRS) score of 5 or more.

### Statistical Analysis

Stata version 12.0 statistical software (StataCorp LP, College Station, Texas) was adopted as statistical software. The risk differences (RDs) or odds ratios (ORs) with the corresponding 95% confidence intervals (95% CIs) were used as measures of the treatment effect of protamine. We accessed the heterogeneity with the Higgins *I*-square (*I* ^2^), which indicated the percentage of the observed between-study viability. *I* ^2^ over 25% and less than 75% was considered as moderately heterogeneous or significant heterogeneity. If *I* ^2^ was under 50%, the endpoint item was considered to be homogeneous, and we ran a meta-analysis by using a fixed-effect model according to the Cochrane Handbook for Systematic Reviews of Interventions. Otherwise, the random-effect model was performed.

### Quality of Evidence Assessment

We used the guidance from the Grading of Recommendations Assessment, Development and Evaluation (GRADE) working group to assess the quality of evidence for the primary outcome (Li et al., 2019). The GRADE summary of findings table was produced using the GradePRO software.

## Results

### Search Result

**FIGURE 1:**
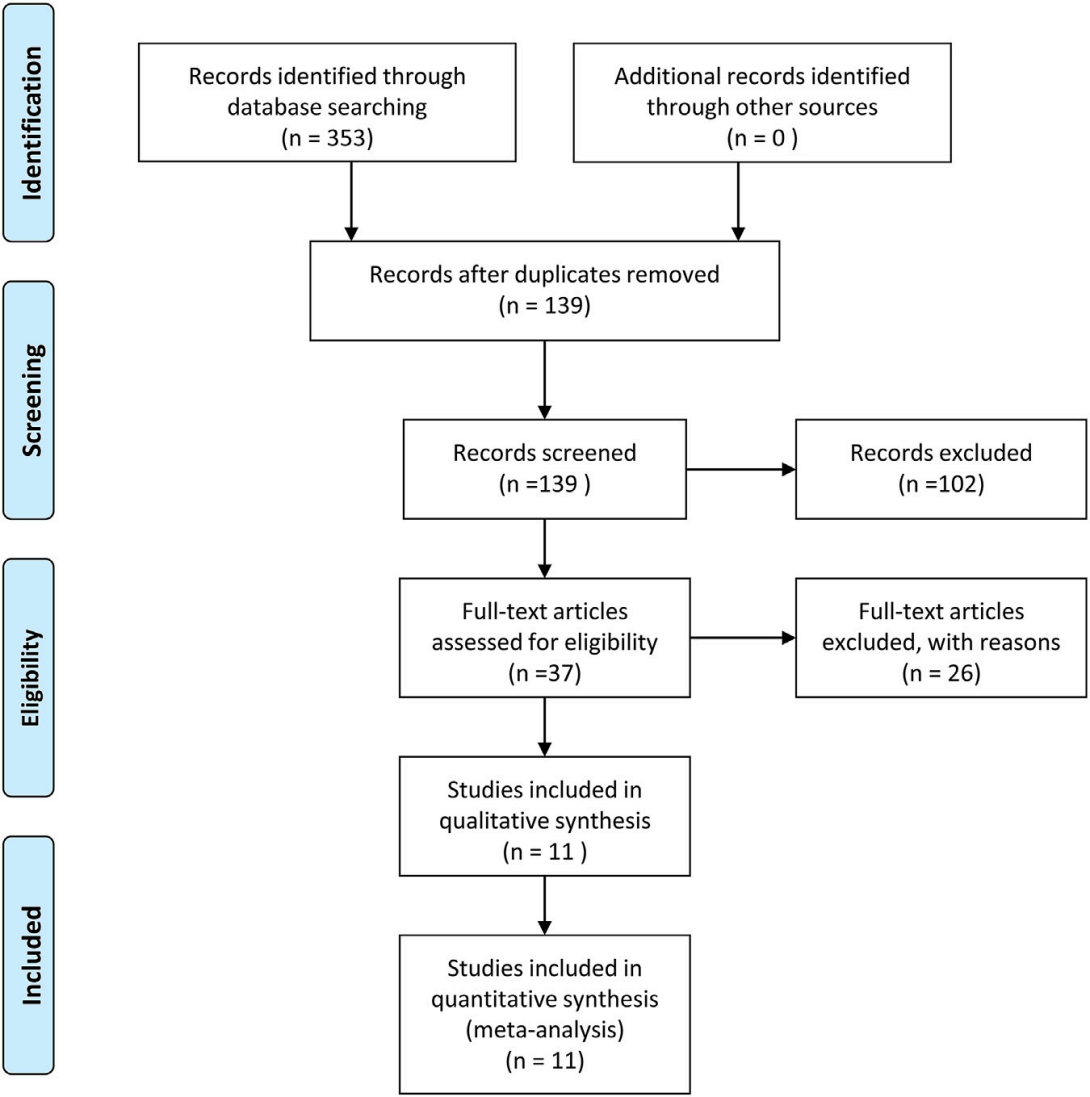
Flowchart of the study selection process.

The screening process is displayed in Figure 1, which is based on the inclusion and exclusion criteria. The search initially yielded a total of 353 articles. After the exclusion of duplicated or irrelevant articles, 139 eligible studies were enrolled in this study. Later, after evaluating the full text of the remaining articles, 37 articles met our inclusion criteria. Finally, 11 studies were involved in our quantitative synthesis.

### Characteristics of Included Studies

Detailed characteristics of the 11 observational articles, including 94,618 participants (median sample size, 1,495; range, 64 to 77,315) with an average age of 76 years (range, 59.1 to 82.6), are listed in Table 2. The majority of studies were performed in the United States (Liang et al., 2021; Treiman et al., 1990; Stone et al., 2010; Stone et al., 2020; McDonald et al., 2013; Liang et al., 2020). Two were in the United Kingdom (Fearn et al., 1997; Dellagrammaticas et al., 2008), and another one was from Italy (Mazzalai et al., 2014). There were 9 Non-RCTs (Randomized Controlled Trials) (Liang et al., 2021; Treiman et al., 1990; Stone et al., 2010; Stone et al., 2020; McDonald et al., 2013; Liang et al., 2020; Mazzalai et al., 2014; Mauney et al., 1995) (*n* = 92,447) and 2 RCTs (Fearn et al., 1997; Dellagrammaticas et al., 2008) involving 2,171 patients randomized to either protamine of heparin or not restrictedly undergoing CEA. In the protamine group, out of the 60,947 patients, 57,148 were allocated to CEA, and 3,799 were from CAS, with an average age from 65.9 to 79.1 years old. In the no protamine group, 57.8% of enrolled participants were from CEA. Nearly all surgeries considered age, whereas only McDonald et al. (2013) ignored this.

**TABLE 2:** Characteristics of publication year, country, study type, cases, general anesthesia, and mean age in each group for included studies.

Study	Years	Country	Study design	General anesthesia	Sample size		Mean age (Years)	
					Protamine	No protamine	Protamine	No protamine
**Protamine use in carotid endarterectomy (CEA)**								
Treiman et al.	1990	United States	Non-RCT	100%	328	369	71	71
Mauney et al.	1995	United States	Non-RCT	98.3%	193	155	65.9	68.8
Fearn et al.	1997	United Kingdom	RCT	100%	31	33	66	61.9
Levison, et al.	1999	United States	Non-RCT	NA	365	42	70.6	69
Dellagrammaticas et al.	2008	United Kingdom	RCT	50%	594	1,513	70	70.4
Stone et al.	2010	United States	Non-RCT	50%	2,087	2,500	69.2	70
Mazzalai et al.	2014	Italy	Non-RCT	100%	201	1,294	75.7	75.1
Stone et al.	2020	United States	Non-RCT l	100%	53,349	23,966	70.0 ± 9.1	69.1 ± 9.2
**Protamine use in carotid artery stenting (CAS)**								
Mcdonald et al.	2013	United States	Non-RCT	NA	555	555	NA	NA
Liang et al.	2020	United States	Non-RCT	NA	944	944	72.7 ± 9.7	73.2 ± 9.4
Liang et al.	2021	United States	Non-RCT	NA	2,300	2,300	70.6 ± 9.5	70.4 ± 9.7

Note: NA: not available; RCT, randomized controlled trials.

### Quality Assessment

Methodological quality and risk of bias in the included observational studies are assessed by two reviewers independently by using the Newcastle-Ottawa Quality Assessment Scale (Mitchell-Jones et al., 2017), which consists of three main categories: selection, comparability, and outcome, with questions in each area corresponding to the study quality (Newhall et al., 2016). The evaluation scores for all non-RCT are listed in Table 3 with the highest quality of 9 points. Studies that scored lower than 5 points equate to low quality, and a score of 6–7 points is regarded as moderate quality. Additionally, the Cochrane Collaboration’s tool is used for assessing the risk of bias of each included RCT. The results of the quality assessment of RCT are provided in Table 4.

**TABLE 3:** The quality assessment in randomized controlled trials.

Author, year	Design	Newcastle-Ottawa scale (NOS)			
		Selection	Comparability	Exposure	Total score
Treiman et al. 1990	Non-RCT	3	1	3	7
Mauney et al. 1995	Non-RCT	3	2	3	8
Levison, et al. 1999	Non-RCT	3	2	2	7
Stone et al. 2010	Non-RCT	3	2	2	7
Mazzalai et al. 2014	Non-RCT	4	1	3	8
Stone et al. 2020	Non-RCT	4	2	2	8
Mcdonald et al. 2013	Non-RCT	3	2	3	8
Liang et al. 2020	Non-RCT	3	1	3	7
Liang et al. 2021	Non-RCT	4	2	2	8

Note: NOS , Newcastle-Ottawa scale.

**TABLE 4:** Cochrane Collaboration’s tool for quality assessment in randomized controlled trials.

Trials	Sequence generation	Allocation concealment	Blinding of outcome assessors	Incomplete outcome data	Selective outcome reporting	Others
Fearn et al. 1997	Low	Unclear	Low	Low	Low	Low
Dellagrammaticas et al. 2008	Low	Low	Low	Low	Low	Unclear

### The Outcome of the Meta-Analysis

There were nearly ten thousand participants, 92% of whom have been undergoing CEA and 8% had been treated with CAS. The detailed results and GRADE assessment of outcomes are shown in Table 5.

**TABLE 5:** The post-operative outcomes of this meta-analysis. The bold values refer to *p*-value < 0.05.

Outcomes	Study numbers	Event rates		Overall effect			Heterogeneity		EQ
		Protamine	No protamine	Effect estimates	95% CIs	*p*-Value	*I* ^2^ (%)	*p*-Value	(GRADE)
**The use of protamine in carotid recanalization**									
Wound hematoma (WH)	4	57/1,488 (3.83%)	305/3,218 (9.48%)	OR (0.268)	0.093–0.774	**0.015**	77.2	0.004	Low
WH requiring re-operation	8	409/60,013 (0.68%)	591/32,714 (1.81%)	OR (0.475)	0.282–0.798	**0.005**	77.3	0.000	Low
WH with hypertension	3	170/1,471 (11.56%)	347/2,607 (13.31%)	OR (0.704)	0.358–1.388	0.311	76.0	0.015	Low
Transient Ischemic Attacks	5	50/4,193 (1.19%)	91/5,248 (1.73%)	OR (0.793)	0.546–1.151	0.222	44.4	0.126	Low
Myocardial Infarction	7	430/60,030 (0.72%)	245/33,072 (0.74%)	OR (0.935)	0.797–1.096	0.408	0.0	0.446	High
Post-operative Stroke	10	735/60,916 (1.21%)	426/33,638 (1.27%)	OR (1.071)	0.944–1.214	0.286	30.1	0.168	Low
Post-operative Death	7	138/56,638 (0.24%)	82/30,526 (0.36%)	RD (0.000)	−0.001–0.001	0.877	0.0	0.719	Low
**The use of protamine in CEA**									
Wound hematoma (WH)	4	57/1,488 (3.83%)	305/3,218 (9.48%)	OR (0.268)	0.093–0.774	**0.015**	77.2	0.004	Low
WH requiring re-operation	6	379/56,769 (0.67%)	546/29,470 (1.85%)	OR (0.429)	0.265–0.694	**0.001**	61.0	0.025	Low
WH with hypertension	2	22/527 (4.17%)	209/1,663 (12.57%)	OR (0.333)	0.057–1.959	0.224	67.6	0.079	Low
Transient Ischemic Attacks	2	3/394 (0.76%)	41/1,449 (2.83%)	OR (0.255)	0.068–0.947	**0.041**	0.0	0.366	High
Myocardial Infarction	4	399/56,231 (0.71%)	222/29,273 (0.76%)	OR (0.902)	0.764–1.065	0.224	0.0	0.661	High
Post-operative Stroke	7	641/57,117 (1.12%)	354/29,839 (1.19%)	OR (1.029)	0.897–1.180	0.687	37	0.146	Low
Post-operative Death	4	106/52,839 (0.20%)	54/26,727 (0.20%)	RD (0.000)	−0.001–0.001	0.878	0.0	0.967	High

Note. CIs, confidence intervals; RD, risk difference; OR, odds ratio; EQ , evidence quality.

### Wound Hematoma

**FIGURE 2:**
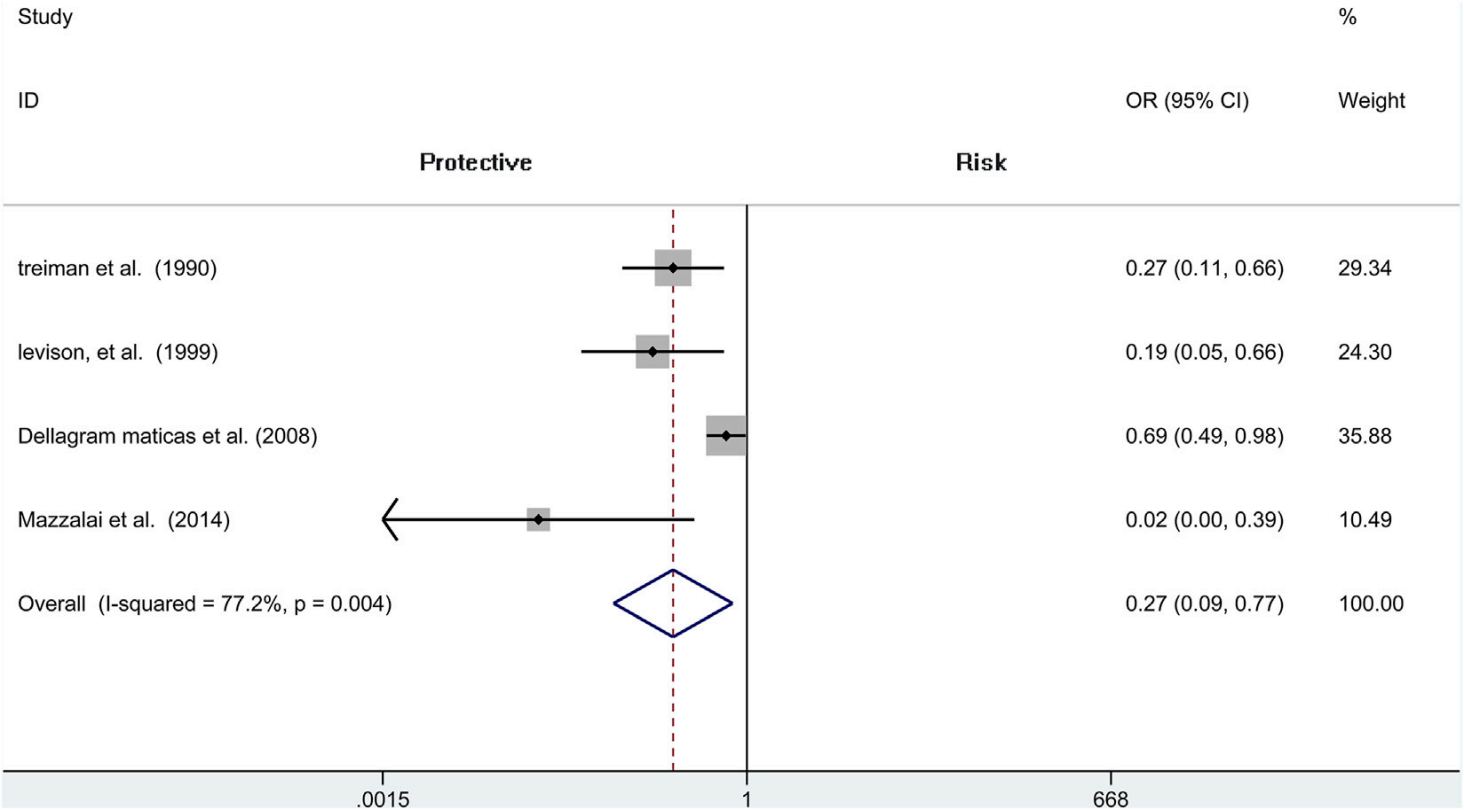
Forest plot for meta-analysis of the incidence of wound hematoma.

We include four independent pieces of research of CEA with 4,706 patients (1,488 of protamine and 3,218 of no-protamine). Among these studies, the incidence of wound hematoma in the protamine group is 3.8% (57 of 1,488), which is smaller than the no-protamine group (9.5%, 305 of 3,218). This comparison fully indicates that the group of protamine is associated with a significantly lower incidence of wound hematoma than participants treated with non-protamine (OR = 0.268, 95% CI = 0.093 to 0.774, *p* = 0.015, Figure 2). Similarly, in the subgroup of CEA, the results are the same. However, a significant heterogeneity was observed (*I* ^2^ = 77.2%, *p* = 0.004). A sensitivity analysis was performed to reveal that the heterogeneity was decreased by deleting the study conducted by Dellagrammaticas et al. (*I* ^2^ = 51.6%, *p* = 0.127).

### Hematoma Requiring Re-operation

**FIGURE 3:**
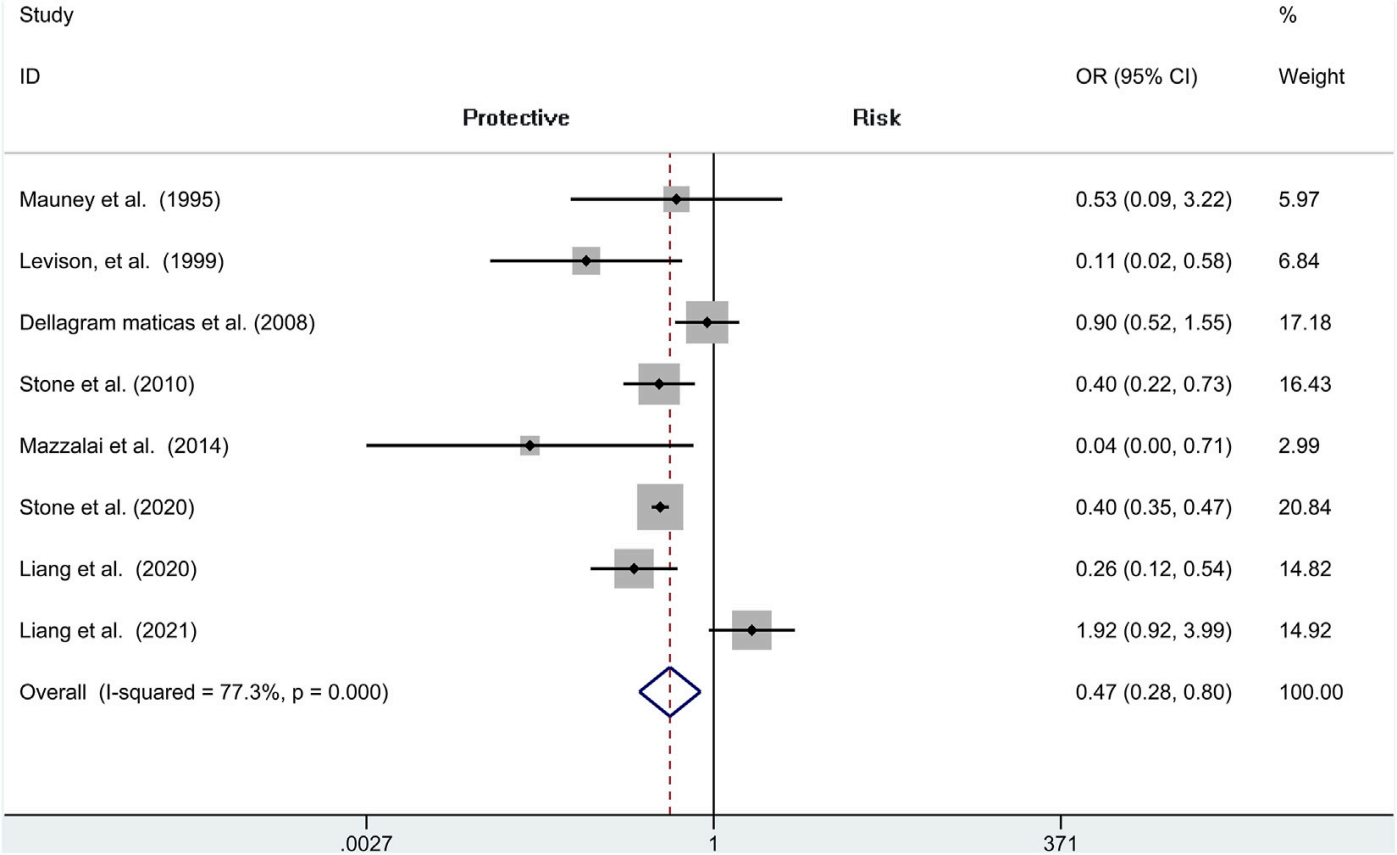
Forest plot for meta-analysis of the incidence of hematoma requiring re-operation.

**FIGURE 4:**
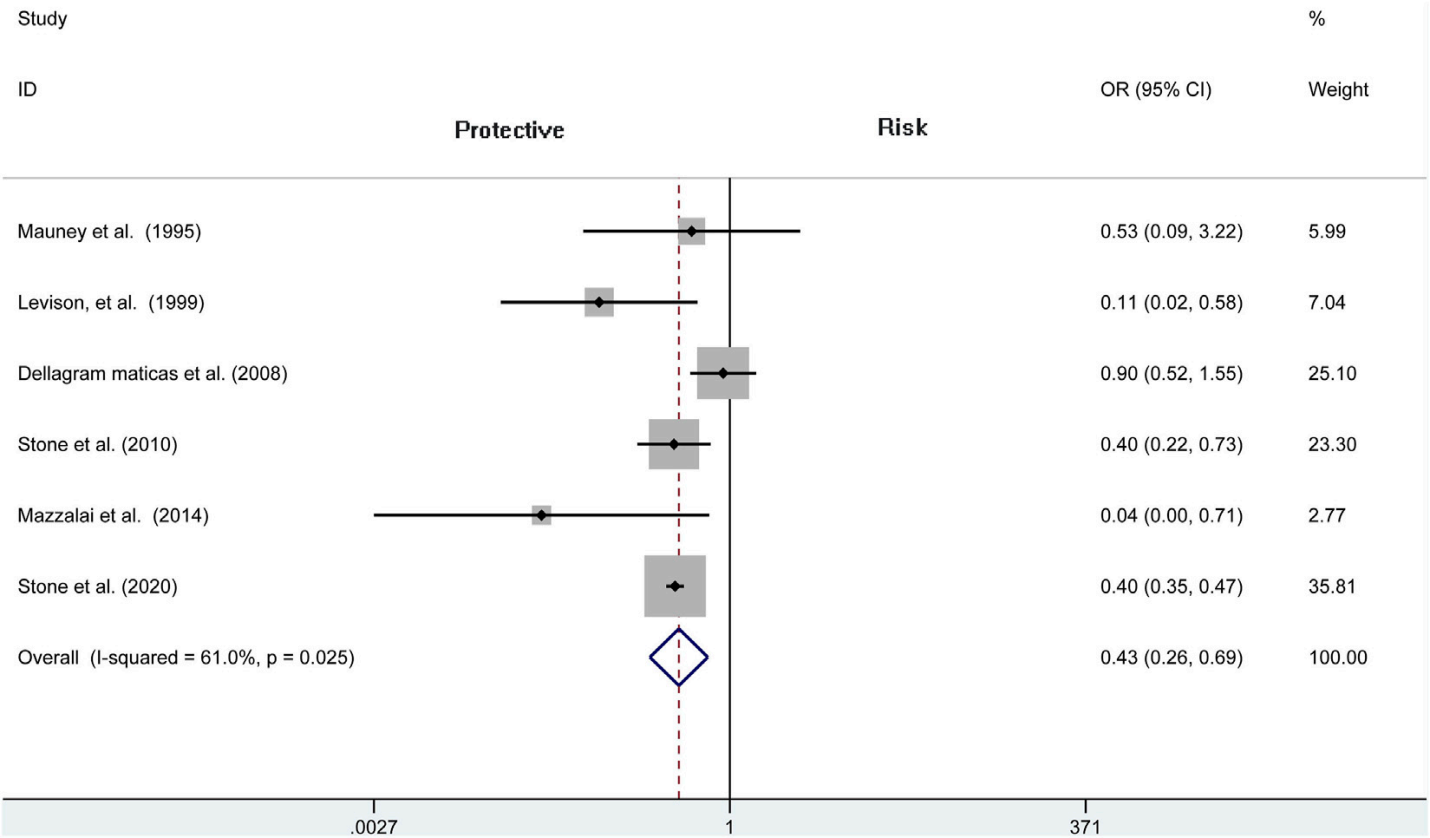
Forest plot for meta-analysis of the incidence of hematoma requiring re-operation in the subgroup of CEA.

Analysis of risk of hematoma requiring re-operation between the protamine and no-protamine groups is provided in eight studies. The proportion estimated in protamine and no-protamine groups is 0.7% (409 of 60,013) versus 1.8% (591 of 32,714). However, a significant heterogeneity was observed, and a random effects model was used (*I* ^2^ = 77.3%, *p* < 0.001). A specific OR of 0.475 (95% CI = 0.282 to 0.798, *p* = 0.005; Figure 3) is obtained, suggesting that the incidence of hematoma requiring re-operation is significantly lower than patients without protamine. Given a significant heterogeneity, we conducted a subgroup analysis, and the results showed that the heterogeneity was decreased (*I* ^2^ = 61%, *p* = 0.025) and that there is also a significant difference in the subgroup of CEA between the two groups (OR = 0.429, 95% CI = 0.265 to 0.694, *p* = 0.001; Figure 4). In addition, we perform a sensitivity analysis and found that the heterogeneity was significantly decreased by deleting the study conducted by Dellagrammaticas et al. (*I* ^2^ = 17.7%, *p* = 0.302).

### Wound Hematoma With Hypertension

**FIGURE 5:**
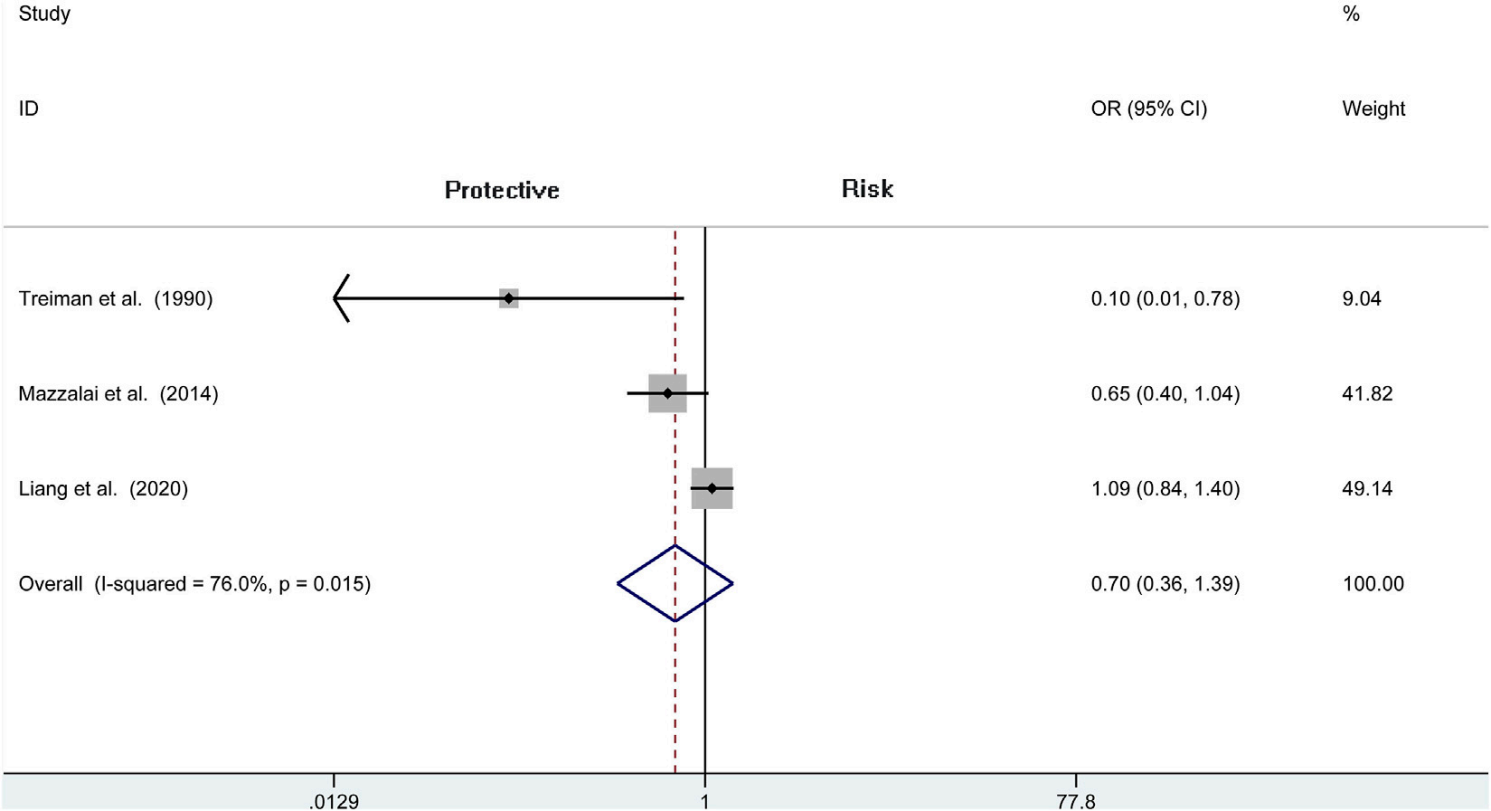
Forest plot for meta-analysis of the incidence of wound hematoma with hypertension.

**FIGURE 6:**
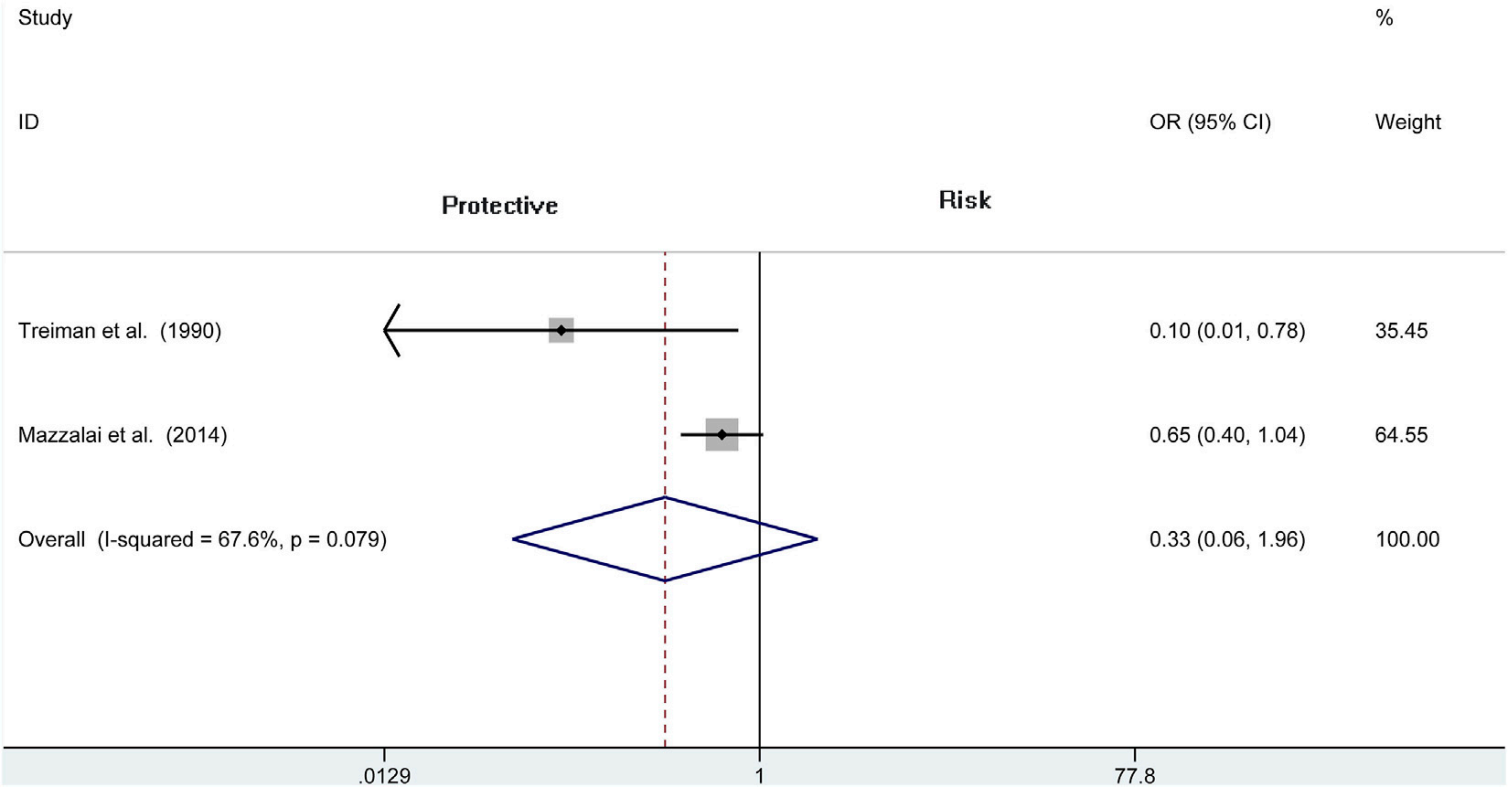
Forest plot for meta-analysis of the incidence of wound hematoma with hypertension in the subgroup of CEA.

Three articles (*N* = 4,078) report the wound hematoma with hypertension. This analysis does not find a significant difference between the two groups (OR = 0.704, 95% CI = 0.358 to 1.388, *p* = 0.311; Figure 5), whereas a high heterogeneity is presented in these studies (*I* ^2^ = 76%, *p* = 0.015). Therefore, we also analyze the subgroup of CEA and reveal that there is no difference among the protamine and no-protamine groups (OR = 0.333, 95% CI = 0.057 to 1.959, *p* = 0.224; Figure 6). However, a high heterogeneity in these studies was also observed (*I* ^2^ = 67.6%, *p* = 0.079). Moreover, we performed a sensitivity analysis, but no significant difference was revealed in the changes of heterogeneity.

### Stroke

**FIGURE 7:**
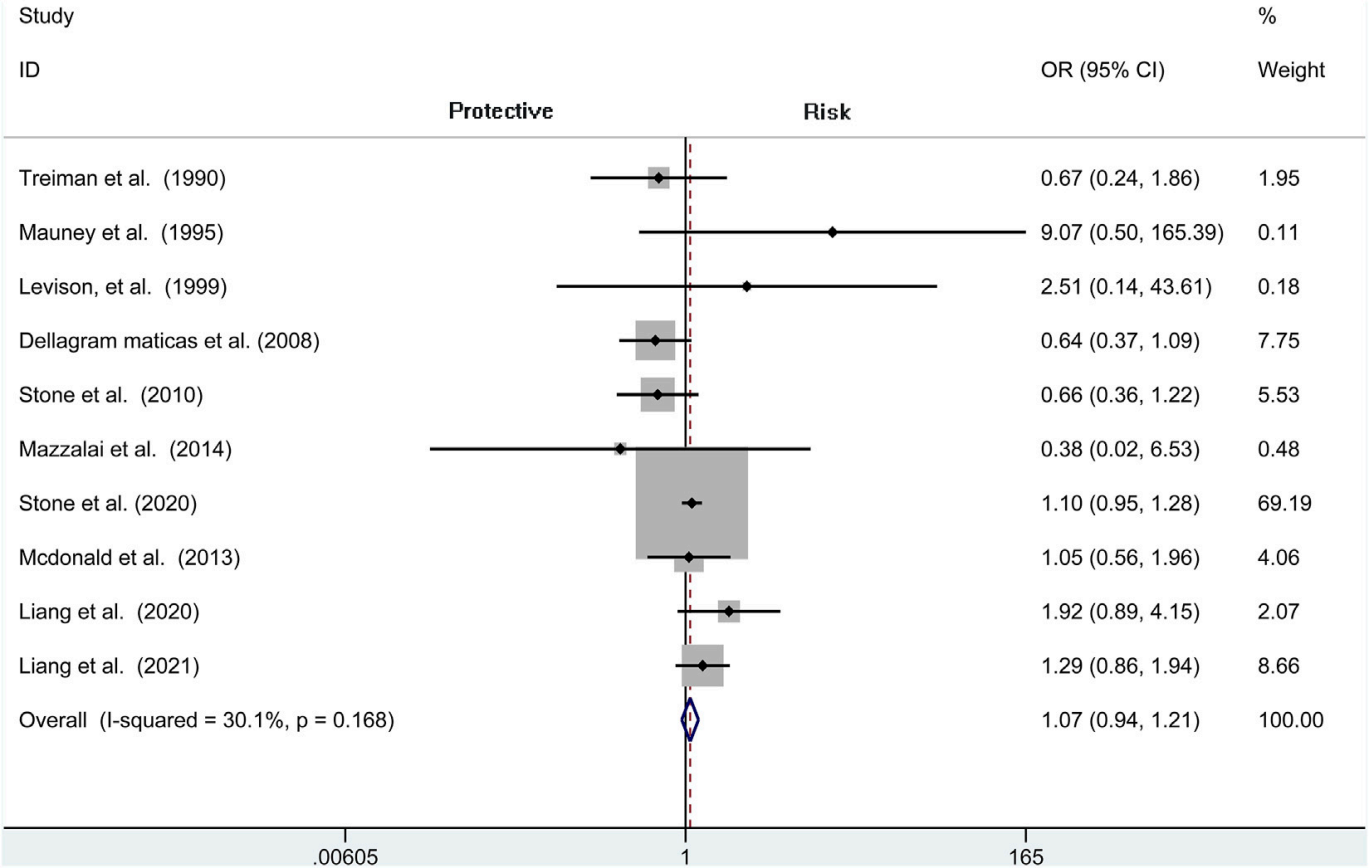
Forest plot for meta-analysis of the incidence of stroke.

**FIGURE 8:**
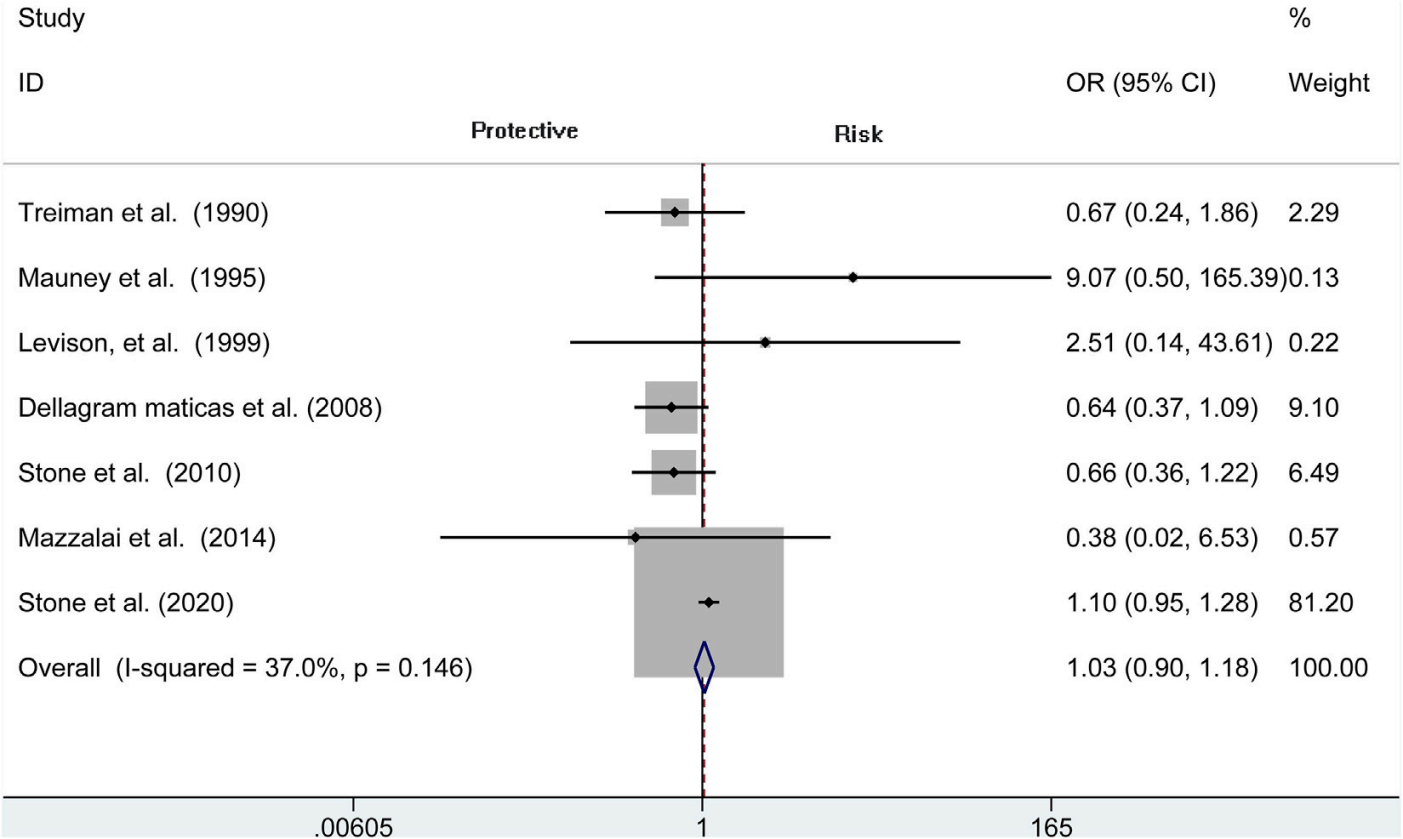
Forest plot for meta-analysis of the incidence of stroke in the subgroup of CEA.

A total of seven independent studies compare protamine with no protamine in participants undergoing CEA, while three studies include participants with carotid stenting. There is no significant heterogeneity among these studies (*I* ^2^ = 30.1%, *p* = 0.168). We think that patients treated with protamine did not have a lower rate of stroke than those treated with no protamine (OR = 1.071, 95% CI = 0.944–1.214, *p* = 0.286, Figure 7). In the subgroup of CEA, there is also no significant difference between the two groups (OR = 1.029, 95% CI = 0.897 to 1.180, *p* = 0.687; Figure 8).

### Transient Ischemic Attacks

**FIGURE 9:**
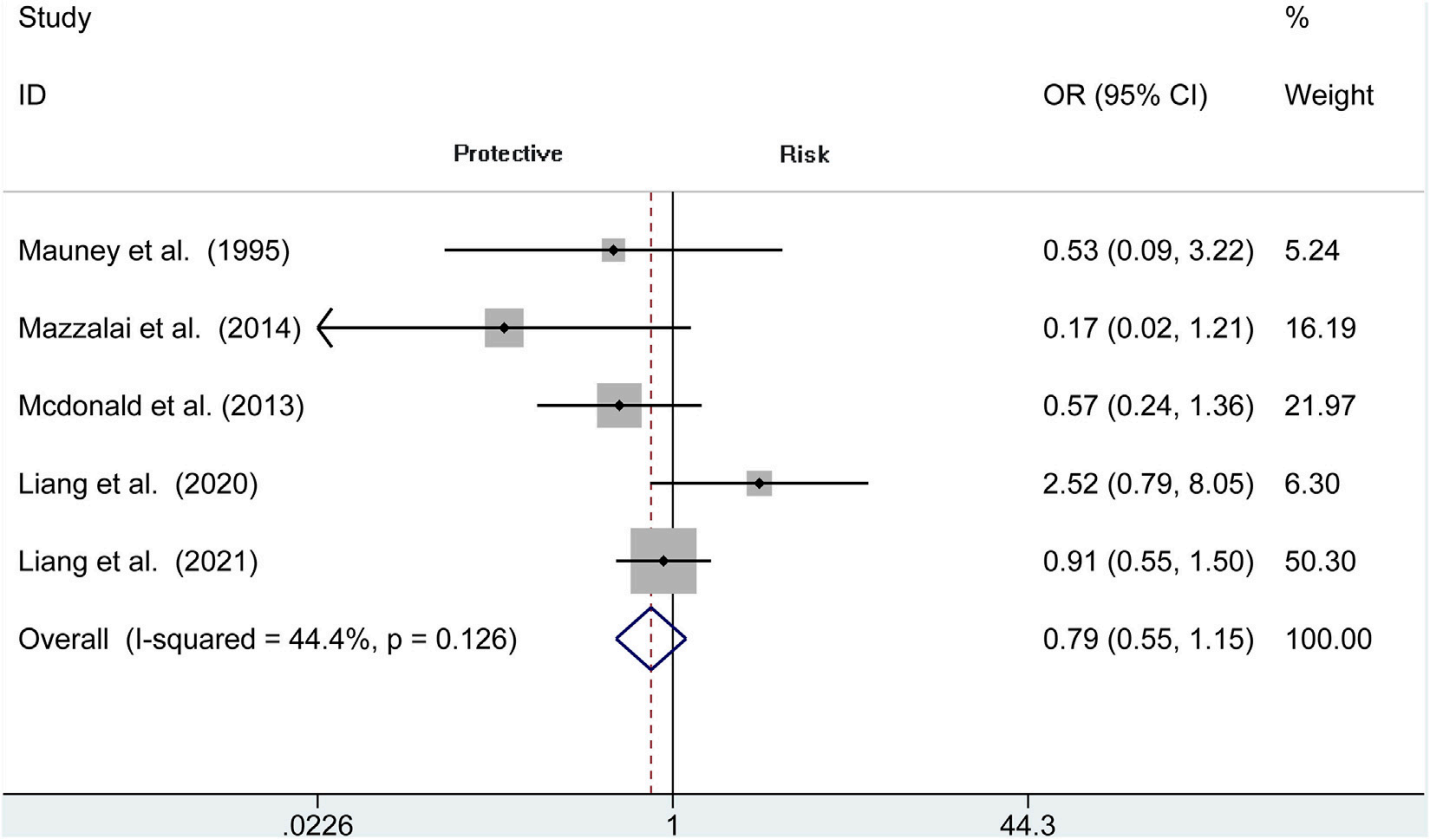
Forest plot for meta-analysis of the incidence of transient ischemic attacks.

The risk of TIA is reported in 5 observational studies (*N* = 9,441). Perioperative TIA occurred in the protamine (50 of 4,193, 1.2%) and no-protamine group (91 of 5,248, 1.7%). No evidence of significant heterogeneity is revealed in these studies (*I* ^2^ = 44.4%, *p* = 0.126). The overall analysis does not prove an apparent difference in TIA rates between protamine and no-protamine groups (OR = 0.793, 95% CI = 0.546 to 1.151, *p* = 0.222; Figure 9). However, a difference is presented in the subgroup of CEA among these two groups (OR = 0.255, 95% CI = 0.068 to 0.947, *p* = 0.041; Supplementary Figure S1).

### Myocardial Infarction (MI)

The MI is reported in seven studies. In these publications, 60,030 and 33,072 patients are enrolled in the protamine and no-protamine groups, respectively. The results reveal that there is no significant difference in occurrence of MI (OR = 0.935, 95% CI = 0.797 to 1.096, *p* = 0.408, Supplementary Figure S2) and between-study heterogeneity is low (*I* ^2^ = 0.0%, *p* = 0.446). Similarly, we find that there is also no difference among protamine and no-protamine patients in the subgroup of CEA (OR = 0.902, 95% CI = 0.764 to 1.065, *p* = 0.224, Supplementary Figure S3).

### Death

Seven publications report post-operative death enrolling 87,164 patients. No significant difference in mortality between the protamine and no-protamine group is seen (RD = 0.000, 95% CI = −0.001 to 0.001, *p* = 0.877, Supplementary Figure S4). The same result is found in the subgroup of CEA as well (RD = 0.000, 95% CI = −0.001 to 0.001, *p* = 0.878, Supplementary Figure S5).

## Discussion

Carotid artery stenosis is a major cause of stroke, which is the most common risk for long-term disability. Surgical treatment (carotid recanalization) is considered significantly meaningful for artery stenosis. Even though it reduces the risk of stroke, it carries a risk of hematoma (Rerkasem et al., 2020). Protamine, which was primarily isolated from salmon fish sperm, is a small, arginine-rich, positively charged protein with similarities to histones in that it has a role in stabilizing DNA in the sperm head (Bakchoul et al., 2016; Boer et al., 2018). It is adopted in a variety of vascular and cardiac procedures to reserve systemic heparin anticoagulation (Phair et al., 2020), especially for carotid recanalization (Lamanna et al., 2019). However, this inevitably leads to bleeding and then further cause major or minor strokes, myocardial infarction, or death (Yuan et al., 2018). Herein, we wonder whether protamine did affect the efficiency and safety of carotid recanalization and try to illustrate its safety and effectiveness in this surgery. Our results demonstrate that protamine can reduce the risk of bleeding without increasing the risk of having other complications.

Reoperation or reintervention is needed if bleeding happened during carotid recanalization, which is associated with the chance of perioperative stroke, MI, or even death. Miklosz et al. (2019) measured the platelet numbers, collagen-induced aggregation, etc. in blood extracted from mice and rats and then furthermore found that protamine has a short-term antiplatelet activity. In 2016, Kakisis et al. (2016) did a meta-analysis showing that the incidence of wound hematoma in the no-protamine group was 6%, whereas only 1.7% happened in the protamine group. They finally indicated that protamine significantly reduced the risk of wound hematoma by 64% without increasing the risk of post-operative stroke. Similarly, our analysis found that the incidence of wound hematoma in the protamine group is 3.8% (57 of 1,488), which is lower than that in the no-protamine group (9.5%, 305 of 3,218). Furthermore, a specific OR of 0.475 (95% CI = 0.282–0.798, *p* = 0.005) was obtained, suggesting that the rates of hematoma requiring re-operation was lower than that in patients without protamine. However, we thought the high rates of wound hematoma may be related to a higher risk of hypertension, but there is no difference presented in the two groups (OR = 0.704, 95% CI = 0.358–1.388, *p* = 0.311). We cannot distinguish whether this result is directly related to protamine use. As far as we know, protamine is a multi-cation strong alkaline polypeptide, which can combine with the glucosaminoglycan of heparin to form a stable complex and inhibit the activity of antithrombin, thus counteracting the anticoagulant effect of heparin and playing the effect of hemostasis. Protamine-induced circulatory changes have been demonstrated by Jastrzebski et al. (Cho et al., 2012), who explicated the role of it by endogenously liberating vasoactive substances.

Cerebral recanalization therapy, either intravenous thrombolysis or mechanical thrombectomy, improves the outcomes of patients with artery stenosis (Zhang et al., 2019), which exerts an increased impact on ischemic diseases. However, some complications involving ischemic injuries such as stroke, TIA, and MI sometimes inevitably followed. We included these three complications in our observations. One study had 365 patients who were subjected to 407 recanalization; 365 (89.6%) received protamine and 42 (10.4%) patients did not; 2.5% (10/407) happened post-operatively in the protamine group. This meta-analysis did not find an association between protamine and stroke (Levison et al., 1999). Likely, in our research, even though the stroke rates were slightly lower than without protamine, our results did not obtain statistical significance (OR = 1.071, 95% CI = 0.944 to 1.214, *p* = 0.286), the same result as TIA. Even though protamine did not affect the incidence of stroke and TIA by multivariate analysis, the risk of MI has also been studied extensively. In light of the 0.7% rate of MI in our study with protamine showing no significant difference (OR = 0.935, 95% CI = 0.797 to 1.096, *p* = 0.408). Furthermore, we should note that the protamine did not change the rate of death among these two groups (RD = 0.000, 95% CI = −0.001 to 0.001, *p* = 0.877). Meanwhile, other studies related to this topic did not find an association between protamine and stroke, TIA, MI, and death. We suspect that there may be a certain stimulation factor of protamine that causes a reduction in bleeding.

Our research has several limitations. The dosage of protamine was not standardized, which may confound the outcomes. Besides the primary inclusion and exclusion criteria, the characteristics of the participants were a little different from each other, potentially causing bias. The number of recent and high-quality studies was too small. Additionally, some heterogeneity was found among included trials due to the different study protocols, patient characteristics, and definitions of clinical endpoints. Moreover, the current study is not registered and there may be a slight deviation, but we strictly followed the procedures of systematic evaluation. Finally, the exact sequence of disease for the included patients cannot be known exactly and protamine might have been administered after a complication occurred rather than before, which might have affected our results.

## Conclusion

This study has crucial implications. The current meta-analysis demonstrates that surgeons should consider routinely using protamine during carotid recanalization especially for CEA, due to the lower incidence of wound hematoma and hematoma requiring re-operation with its use. These findings, however, have inherent limitations, such as obvious heterogeneity and data from retrospective reviews; therefore, they cannot be regarded robust enough to provide a firm recommendation in clinical practice. With regard to the carotid artery stenting, there were fewer studies examining the effect of protamine; herein, further research is necessary to illustrate whether consistent results exist across all types of carotid revascularization.

## Brief statement

Protamine, which was primarily isolated from salmon fish sperm, is a small, arginine-rich, positively charged protein with similarities to histones in that it has a role in stabilizing DNA in the sperm head. Despite it being adopted in a variety of vascular and cardiac procedures to reserve systemic heparin anticoagulation, especially for carotid recanalization, this inevitably leads to bleeding and then further causes major or minor strokes, myocardial infarction, or death. Herein, a comprehensive review and meta-analysis are necessary to illustrate the efficiency of protamine for carotid recanalization. This study demonstrated that protamine was effective in reducing hematoma without increasing the risk of having other complications.

## Data Availability

The original contributions presented in the study are included in the article/Supplementary Material, further inquiries can be directed to the corresponding authors.

## Abbreviations

CEA, Carotid endarterectomy; CAS, Carotid artery stenting; MI, Myocardial Infarction; RDs, Risk differences; ORs, Odds ratios; FDA, Food and Drug Administration; CIs, Confidence Intervals; RCTs, Randomized Controlled Trials; TIA, Transient Ischemic Attacks.

## References

[B1] Abbott A. L. Paraskevas K. I. Kakkos S. K. Golledge J. Eckstein H. H. Diaz-Sandoval L. J. (2015). Systematic Review of Guidelines for the Management of Asymptomatic and Symptomatic Carotid Stenosis. Stroke 46 (11), 3288–3301. 10.1161/STROKEAHA.115.003390 26451020

[B2] Al-Kassou B. Kandt J. Lohde L. Shamekhi J. Sedaghat A. Tabata N. (2020). Safety and Efficacy of Protamine Administration for Prevention of Bleeding Complications in Patients Undergoing TAVR. JACC Cardiovasc. Interv. 13 (12), 1471–1480. 10.1016/j.jcin.2020.03.041 32553337

[B3] Bakchoul T. Jouni R. Warkentin T. E. (2016). Protamine (Heparin)-induced Thrombocytopenia: a Review of the Serological and Clinical Features Associated with Anti-protamine/heparin Antibodies. J. Thromb. Haemost. 14 (9), 1685–1695. 10.1111/jth.13405 27378603

[B4] Boer C. Meesters M. I. Veerhoek D. Vonk A. B. A. (2018). Anticoagulant and Side-Effects of Protamine in Cardiac Surgery: a Narrative Review. Br. J. Anaesth. 120 (5), 914–927. 10.1016/j.bja.2018.01.023 29661409

[B5] Cho Y. D. Lee J. Y. Seo J. H. Kang H. S. Kim J. E. Kwon O. K. (2012). Early Recurrent Hemorrhage after Coil Embolization in Ruptured Intracranial Aneurysms. Neuroradiology 54 (7), 719–726. 10.1007/s00234-011-0950-3 21969241

[B6] Dellagrammaticas D. Lewis S. C. Gough M. J. (2008). Is Heparin Reversal with Protamine after Carotid Endarterectomy Dangerous? Eur. J. Vasc. Endovasc Surg. 36 (1), 41–44. 10.1016/j.ejvs.2008.01.021 18406179

[B7] Dharmakidari S. Bhattacharya P. Chaturvedi S. (2017). Carotid Artery Stenosis: Medical Therapy, Surgery, and Stenting. Curr. Neurol. Neurosci. Rep. 17 (10), 77. 10.1007/s11910-017-0786-2 28825185

[B8] Fearn S. J. Parry A. D. Picton A. J. Mortimer A. J. McCollum C. N. (1997). Should Heparin Be Reversed after Carotid Endarterectomy? A Randomised Prospective Trial. Eur. J. Vasc. Endovasc Surg. 13 (4), 394–397. 10.1016/s1078-5884(97)80082-2 9133992

[B9] Howell S. J. (2007). Carotid Endarterectomy. Br. J. Anaesth. 99 (1), 119–131. 10.1093/bja/aem137 17556351

[B10] Jaques L. B. (1973). Protamine--antagonist to Heparin. Can. Med. Assoc. J. 108 (10), 1291–1297. 4122234 PMC1941450

[B11] Kakisis J. D. Antonopoulos C. N. Moulakakis K. G. Schneider F. Geroulakos G. Ricco J. B. (2016). Protamine Reduces Bleeding Complications without Increasing the Risk of Stroke after Carotid Endarterectomy: A Meta-Analysis. Eur. J. Vasc. Endovasc Surg. 52 (3), 296–307. 10.1016/j.ejvs.2016.05.033 27389942

[B12] Lamanna A. Maingard J. Barras C. D. Kok H. K. Handelman G. Chandra R. V. (2019). Carotid Artery Stenting: Current State of Evidence and Future Directions. Acta Neurol. Scand. 139 (4), 318–333. 10.1111/ane.13062 30613950

[B13] Levison J. A. Faust G. R. Halpern V. J. Theodoris A. Nathan I. Kline R. G. (1999). Relationship of Protamine Dosing with Postoperative Complications of Carotid Endarterectomy. Ann. Vasc. Surg. 13 (1), 67–72. 10.1007/s100169900222 9878659

[B14] Li Q. Gao Y. Xin W. Zhou Z. Rong H. Qin Y. (2019). Meta-Analysis of Prognosis of Different Treatments for Symptomatic Moyamoya Disease. World Neurosurg. 127, 354–361. 10.1016/j.wneu.2019.04.062 30995556

[B15] Liang P. Motaganahalli R. Swerdlow N. J. Dansey K. Varkevisser R. R. B. Li C. (2021). Protamine Use in Transfemoral Carotid Artery Stenting Is Not Associated with an Increased Risk of Thromboembolic Events. J. Vasc. Surg. 73 (1), 142–e4. 10.1016/j.jvs.2020.04.526 32535154

[B16] Liang P. Motaganahalli R. L. Malas M. B. Wang G. J. Eldrup-Jorgensen J. Cronenwett J. L. (2020). Protamine Use in Transcarotid Artery Revascularization Is Associated with Lower Risk of Bleeding Complications without Higher Risk of Thromboembolic Events. J. Vasc. Surg. 72 (6), 2079–2087. 10.1016/j.jvs.2020.02.019 32273225

[B17] Lynch N. P. Kavanagh E. G. (2016). Does Routine Reversal of Heparin with Protamine Sulphate in Patients Undergoing Carotid Endarterectomy Reduce Bleeding Complications without Leading to Increased Thromboembolic Complications? Eur. J. Vasc. Endovasc Surg. 51 (1), 150. 10.1016/j.ejvs.2015.09.001 26482510

[B18] Mauney M. C. Buchanan S. A. Lawrence W. A. Bishop A. Sinclair K. Daniel T. M. (1995). Stroke Rate Is Markedly Reduced after Carotid Endarterectomy by Avoidance of Protamine. J. Vasc. Surg. 22 (3), 264–270. ; discussion 269-70. 10.1016/s0741-5214(95)70140-0 7674469

[B19] Mazzalai F. Piatto G. Toniato A. Lorenzetti R. Baracchini C. Ballotta E. (2014). Using Protamine Can Significantly Reduce the Incidence of Bleeding Complications after Carotid Endarterectomy without Increasing the Risk of Ischemic Cerebral Events. World J. Surg. 38 (5), 1227–1232. 10.1007/s00268-013-2347-4 24276985

[B20] McDonald J. S. Kallmes D. F. Lanzino G. Cloft H. J. (2013). Protamine Does Not Increase Risk of Stroke in Patients with Elective Carotid Stenting. Stroke 44 (7), 2028–2030. 10.1161/STROKEAHA.113.001188 23760211

[B21] Miklosz J. Kalaska B. Kaminski K. Rusak M. Szczubialka K. Nowakowska M. (2019). The Inhibitory Effect of Protamine on Platelets Is Attenuated by Heparin without Inducing Thrombocytopenia in Rodents. Mar. Drugs 17 (9). 10.3390/md17090539 PMC6780366 31533230

[B22] Mitchell-Jones N. Gallos I. Farren J. Tobias A. Bottomley C. Bourne T. (2017). Psychological Morbidity Associated with Hyperemesis Gravidarum: a Systematic Review and Meta-Analysis. BJOG 124 (1), 20–30. 10.1111/1471-0528.14180 27418035

[B23] Newhall K. A. Saunders E. C. Larson R. J. Stone D. H. Goodney P. P. (2016). Use of Protamine for Anticoagulation during Carotid Endarterectomy: A Meta-Analysis. JAMA Surg. 151 (3), 247–255. 10.1001/jamasurg.2015.3592 26501944 PMC13431779

[B24] Patel R. B. Beaulieu P. Homa K. Goodney P. P. Stanley A. C. Cronenwett J. L. (2013). Shared Quality Data Are Associated with Increased Protamine Use and Reduced Bleeding Complications after Carotid Endarterectomy in the Vascular Study Group of New England. J. Vasc. Surg. 58 (6), 1518–e1. 10.1016/j.jvs.2013.06.064 24011737 PMC4279241

[B25] Phair J. Futchko J. Trestman E. B. Carnevale M. Friedmann P. Shukla H. (2020). Protamine Sulfate Use during Tibial Bypass Does Not Appear to Increase Thrombotic Events or Affect Short-Term Graft Patency. Vascular 28 (6), 708–714. 10.1177/1708538120924149 32393108

[B26] Rerkasem A. Orrapin S. Howard D. P. Rerkasem K. (2020). Carotid Endarterectomy for Symptomatic Carotid Stenosis. Cochrane Database Syst. Rev. 9, CD001081. 10.1002/14651858.CD001081.pub4 32918282 PMC8536099

[B27] Setacci C. Sterpetti A. de Donato G. (2018). Introduction: Carotid Endarterectomy versus Carotid Stenting-A Never-Ending story. Semin. Vasc. Surg. 31 (1), 1–3. 10.1053/j.semvascsurg.2018.03.001 29891027

[B28] Sokolowska E. Kalaska B. Miklosz J. Mogielnicki A. (2016). The Toxicology of Heparin Reversal with Protamine: Past, Present and Future. Expert Opin. Drug Metab. Toxicol. 12 (8), 897–909. 10.1080/17425255.2016.1194395 27223896

[B29] Spence J. D. Song H. Cheng G. (2016). Appropriate Management of Asymptomatic Carotid Stenosis. Stroke Vasc. Neurol. 1 (2), 64–71. 10.1136/svn-2016-000016 28959466 PMC5435189

[B30] Spiliopoulos S. Vasiniotis Kamarinos N. Reppas L. Palialexis K. Brountzos E. (2019). Carotid Artery Stenting: an Update. Curr. Opin. Cardiol. 34 (6), 616–620. 10.1097/HCO.0000000000000679 31436557

[B31] Stone D. H. Giles K. A. Kubilis P. Suckow B. D. Goodney P. P. Huber T. S. (2020). Protamine Reduces Serious Bleeding Complications Associated with Carotid Endarterectomy in Asymptomatic Patients without Increasing the Risk of Stroke, Myocardial Infarction, or Death in a Large National AnalysisStroke, Myocardial Infarction, or Death in a Large National Analysis. Eur. J. Vasc. Endovasc Surg. 60 (6), 800–807. 10.1016/j.ejvs.2020.08.047 33127243

[B32] Stone D. H. Nolan B. W. Schanzer A. Goodney P. P. Cambria R. A. Likosky D. S. (2010). Protamine Reduces Bleeding Complications Associated with Carotid Endarterectomy without Increasing the Risk of Stroke. J. Vasc. Surg. 51 (3), 559–e1. 564 e1. 10.1016/j.jvs.2009.10.078 20045609 PMC5240820

[B33] Treiman R. L. Cossman D. V. Foran R. F. Levin P. M. Cohen J. L. Wagner W. H. (1990). The Influence of Neutralizing Heparin after Carotid Endarterectomy on Postoperative Stroke and Wound Hematoma. J. Vasc. Surg. 12 (4), 440–446. 10.1016/0741-5214(90)90046-d 2214039

[B34] Yip H. K. Sung P. H. Wu C. J. Yu C. M. (2016). Carotid Stenting and Endarterectomy. Int. J. Cardiol. 214, 166–174. 10.1016/j.ijcard.2016.03.172 27061654

[B35] Yuan G. Zhou S. Wu W. Zhang Y. Lei J. Huang B. (2018). Carotid Artery Stenting versus Carotid Endarterectomy for Treatment of Asymptomatic Carotid Artery Stenosis. Int. Heart J. 59 (3), 550–558. 10.1536/ihj.17-312 29681577

[B36] Zhang Z. Pu Y. Mi D. Liu L. (2019). Cerebral Hemodynamic Evaluation after Cerebral Recanalization Therapy for Acute Ischemic Stroke. Front. Neurol. 10, 719. 10.3389/fneur.2019.00719 31333570 PMC6618680

