# Supplementary material for: A Meta-Analysis of Using Protamine for Reducing the Risk of Hemorrhage During Carotid Recanalization: Direct Comparisons of Post-operative Complications
Source: Front Pharmacol. 2022 Feb 25;13:796329. doi: 10.3389/fphar.2022.796329 (PMC8914204; doi:10.3389/fphar.2022.796329)
Supplement: Supplementary file 1 [file DataSheet1.docx]

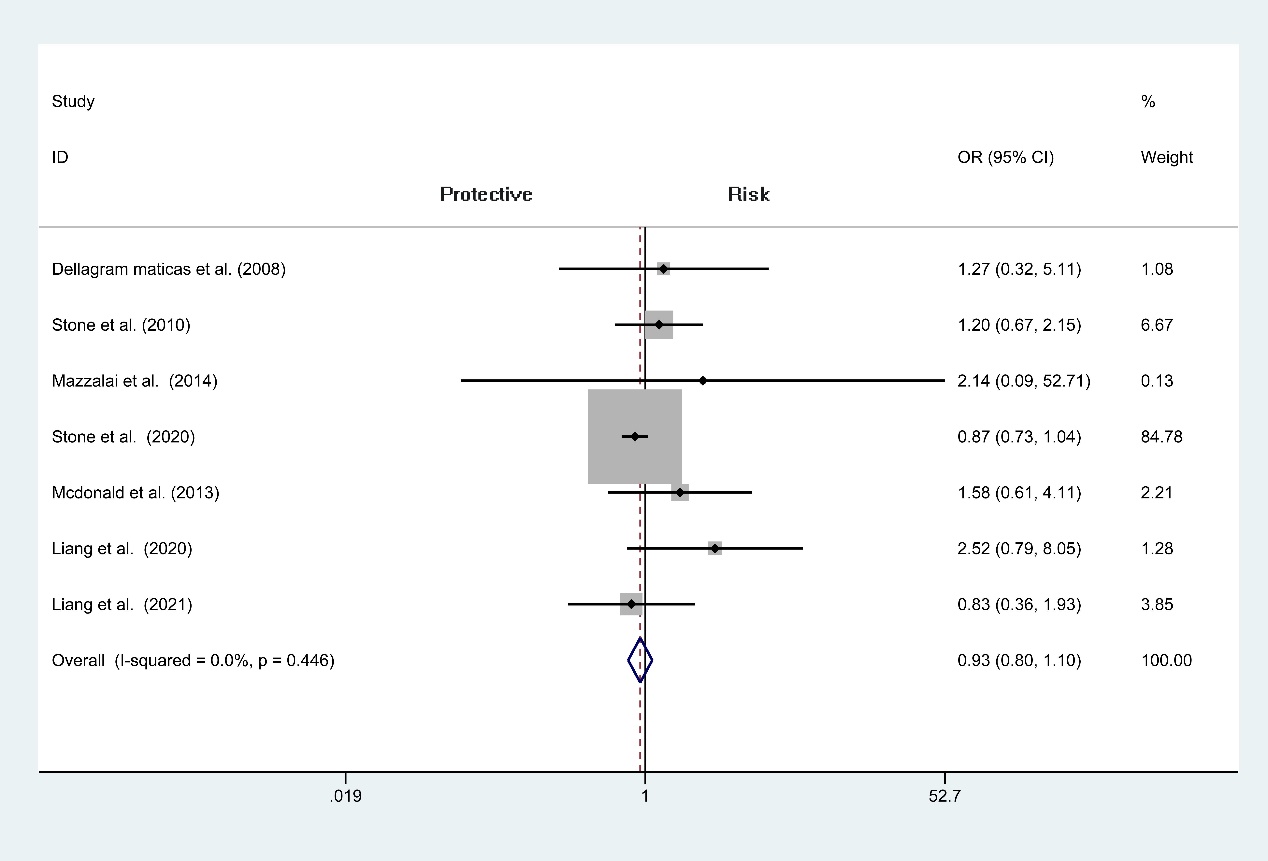


sFigure1. Forest plot for meta-analysis of the incidence of transient ischemic attacks in the subgroup of CEA.


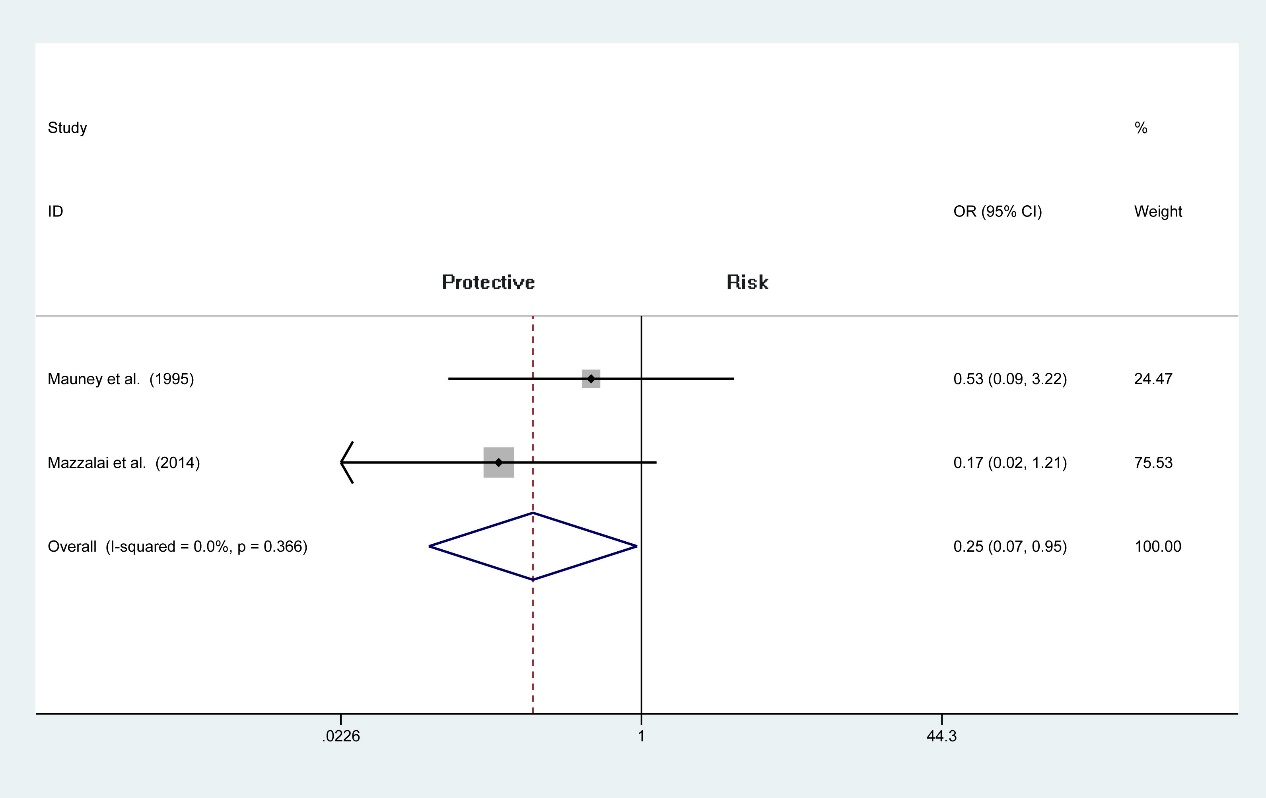


sFigure2. Forest plot for meta-analysis of the incidence of myocardial infarction.


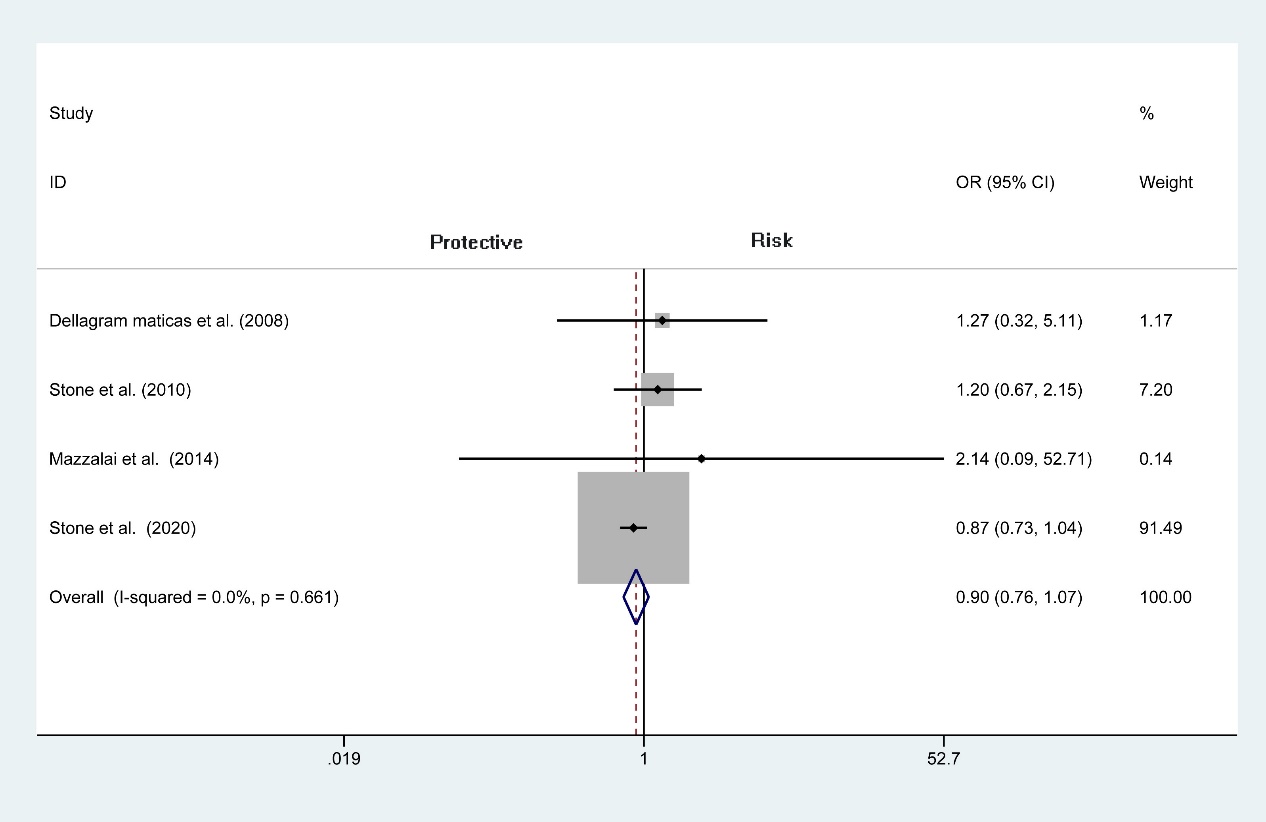


sFigure3. Forest plot for meta-analysis of the incidence of myocardial infarction in the subgroup of CEA.


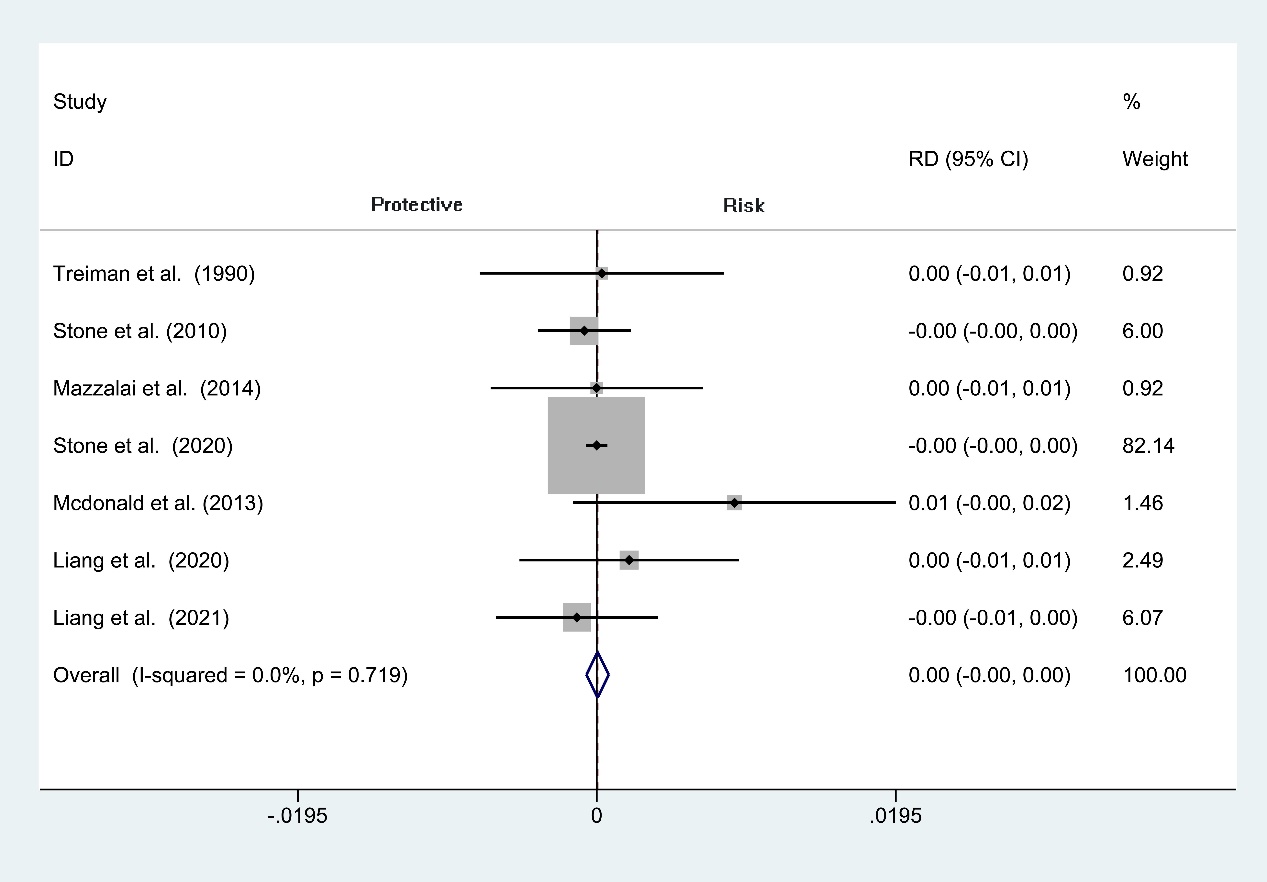


sFigure4. Forest plot for meta-analysis of the incidence of death.


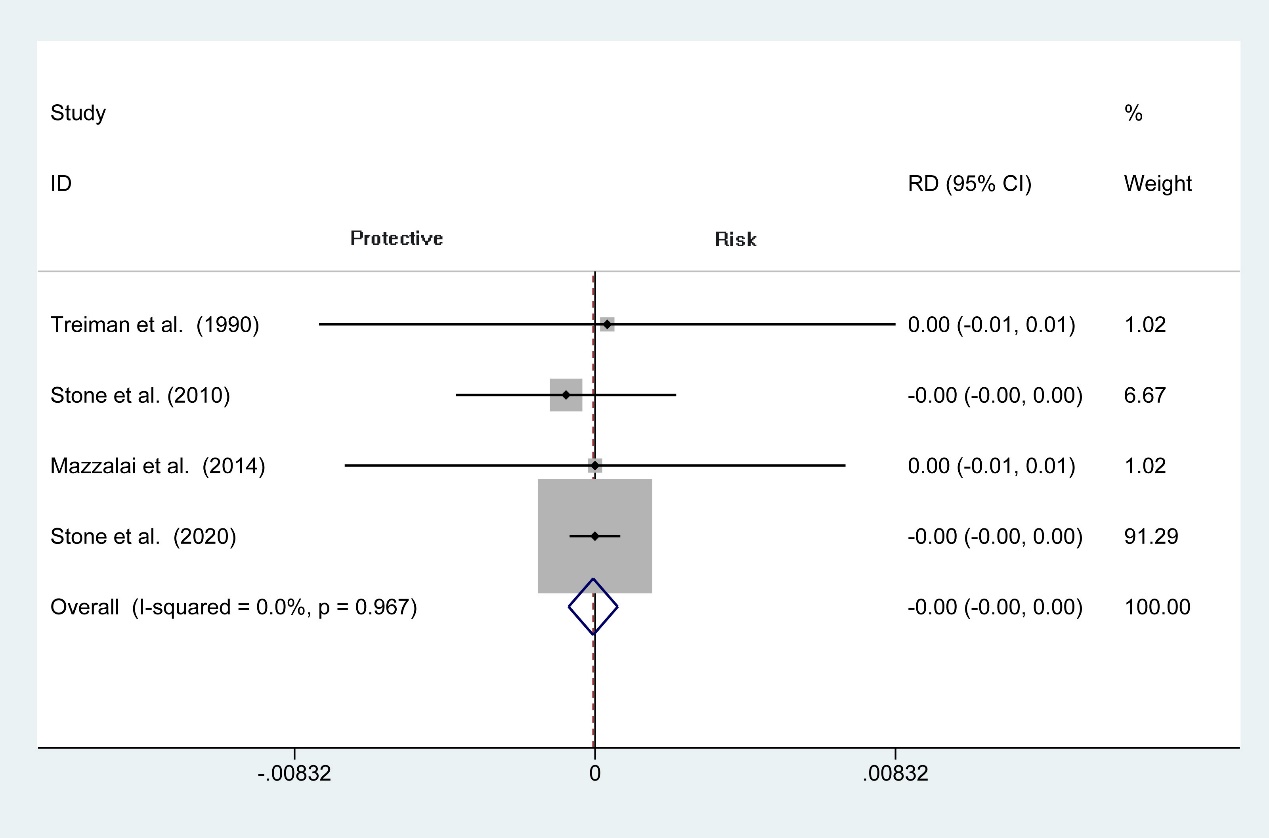


sFigure5. Forest plot for meta-analysis of the incidence of death in the subgroup of CEA.
